# Phenotype-Specific Outcome and Treatment Response in Heart Failure with Preserved Ejection Fraction with Comorbid Hypertension and Diabetes: A 12-Month Multicentered Prospective Cohort Study

**DOI:** 10.3390/jpm13081218

**Published:** 2023-07-31

**Authors:** Ngoc-Thanh-Van Nguyen, Hoai-An Nguyen, Hai Hoang Nguyen, Binh Quang Truong, Hoa Ngoc Chau

**Affiliations:** 1 Department of Internal Medicine, University of Medicine and Pharmacy at Ho Chi Minh City, Ho Chi Minh City 700000, Vietnam; vanntnguyen.md@ump.edu.vn; 2 Cardiovascular Department, Nhan Dan Gia Dinh Hospital, Ho Chi Minh City 700000, Vietnam; 3 Cardiovascular Center, University Medical Center, Ho Chi Minh City 700000, Vietnam; 4 Central Clinical School, Monash University, Melbourne, VIC 3004, Australia

**Keywords:** heart failure with preserved ejection fraction, hypertension, diabetes, phenotype, personalized approach, all-cause mortality, heart failure hospitalization

## Abstract

Despite evidence of SGLT2 inhibitors in improving cardiovascular outcomes of heart failure with preserved ejection fraction (HFpEF), the heterogenous mechanism and characteristic multimorbidity of HFpEF require a phenotypic approach. Metabolic phenotype, one common HFpEF phenotype, has various presentations and prognoses worldwide. We aimed to identify different phenotypes of hypertensive-diabetic HFpEF, their phenotype-related outcomes, and treatment responses. The primary endpoint was time to the first event of all-cause mortality or hospitalization for heart failure (HHF). Among 233 recruited patients, 24.9% experienced primary outcomes within 12 months. A total of 3.9% was lost to follow-up. Three phenotypes were identified. Phenotype 1 (n = 126) consisted of lean, elderly females with chronic kidney disease, anemia, and concentric hypertrophy. Phenotype 2 (n = 62) included younger males with coronary artery disease. Phenotype 3 (n = 45) comprised of obese elderly with atrial fibrillation. Phenotype 1 and 2 reported higher primary outcomes than phenotype 3 (*p* = 0.002). Regarding treatment responses, SGLT2 inhibitor was associated with fewer primary endpoints in phenotype 1 (*p* = 0.003) and 2 (*p* = 0.001). RAAS inhibitor was associated with fewer all-cause mortality in phenotype 1 (*p* = 0.003). Beta blocker was associated with fewer all-cause mortality in phenotype 1 (*p* = 0.024) and fewer HHF in phenotype 2 (*p* = 0.011). Our pioneering study supports the personalized approach to optimize HFpEF management in hypertensive-diabetic patients.

## 1. Introduction

Heart failure with preserved ejection fraction (HFpEF) has been a pressing issue in recent years, accounting for approximately 50% of heart failure population [1,2]. Increasing age, cumulative comorbidity, and diagnostic advancement are considered the main drivers for the growing prevalence and incidence of HFpEF worldwide [3]. Regardless of a seemingly normal ejection fraction (EF ≥ 50%), a recent real-world registry revealed a striking 75% five-year mortality rate in HFpEF patients, which was similar to those with EF < 40% [4].

Unlike heart failure with reduced ejection fraction (HFrEF) with four life-saving therapies, sodium–glucose cotransporter-2 (SGLT2) inhibitor is the only disease-modifying medication with a class IIa indication for HFpEF, thanks to the success of EMPEROR-Preserved (2021) and DELIVER (2022) trials [5,6,7]. The efficacy of other class IIb therapies was mostly limited to specific subgroups of patients, such as males with low EF range (TOPCAT) or females with EF below 57% (PARAGON-HF) [5,8,9]. This partially reflects the heterogenous nature of HFpEF, which does not limit to the underlying pathologic processes but also manifests as diverse clinical courses and treatment responses [10,11,12,13].

One widely acknowledged HFpEF mechanism is chronic inflammation caused by co-occurring conditions [10,14]. These diseases cluster together to form specific phenotypes, offering further guidance on treatment [15,16,17]. Because HFpEF is notoriously known for non-cardiovascular mortality, apart from SGLT2 inhibitor, other therapies are often required to optimize comorbid conditions [18]. Specifically, the comorbidity-based phenotyping offers further guidance on treatment choice [11,13,15,16].

Since its first introduction in 2014, the phenotypic approach has witnessed a tremendous transformation, shifting its focus from describing phenotype-specific clinical presentations to predicting phenotype-related outcomes and treatment responses [11,12,13,19,20]. A variety of techniques exist for the identification of phenotypes, from employing basic demographic and comorbidity burden to integrating complex clinical and biomarker characteristics [21]. Regardless of the phenotyping process, some ubiquitous phenotypes emerge, including the metabolic one, typified by diabetes, obesity, and insulin resistance [15,16,19,22,23,24].

Recent analyses from American, European (I-PRESERVE, CHARM-Preserved), and Asian (ASIAN-HF) trials described a non-obese metabolic population with different prognoses, suggesting the existence of diverse phenotypes within the same metabolic umbrella [20,25]. Among all metabolic phenotypes in HFpEF, hypertension is the most common comorbidity. The prevalence of hypertension in HFpEF ranges from 71% in the ASIAN-HF registry to 91% in the TOPCAT trial [11,25]. This prevalence was further heightened in metabolic phenotypes, at 95% in the ASIAN-HF registry and 92% in the TOPCAT trial, reflecting both the rising co-prevalence of hypertension and diabetes as well as their co-predictive nature, especially in HFpEF patients [11,25].

In Vietnam, we conducted a multicentered HFpEF study in 2021, identifying four different phenotypes, two of which had the highest and almost similar prevalence of hypertension and diabetes [26]. Given the growing co-prevalence and diverse prognosis of hypertensive-diabetic HFpEF in recent trials, we conducted a pioneering study in the Vietnamese population with three specific aims: (1) to identify different phenotypes in hypertensive-diabetic HFpEF patients, (2) to compare their 12-month composite outcome of time to the first event of all-cause mortality or hospitalization for heart failure (HHF), and (3) to evaluate the treatment response of each phenotype to medications, including SGLT2 inhibitor, renin–angiotensin–aldosterone system (RAAS) inhibitor, beta blocker and mineralocorticoid receptor antagonist (MRA).

## 2. Materials and Methods

### 2.1. Patient Population

The study was carried out at two teaching university hospitals: University Medical Center, Ho Chi Minh city and Nhan Dan Gia Dinh Hospital. These two hospitals followed the same heart failure management protocol issued by the Ministry of Health of Vietnam in 2020. We initially planned to recruit patients from January to April 2021 and follow them for 12 months. However, due to COVID-19, the recruitment was extended until April 2022, and the last follow-up ended in February 2023. The study received approval from the Ethics Committee of Biomedical Research at the University of Medicine and Pharmacy at Ho Chi Minh city (number: 2129-ĐHYD) and was registered on Clinicaltrial.gov (identifier number: NCT04835194). Individual informed consent was collected before data collection.

*Inclusion criteria:*

• Outpatient individuals, ≥18 years old;
• Previously diagnosed with or currently treated for type 2 diabetes mellitus;
• Previously diagnosed with or currently treated for primary hypertension;
• Preexisting or newly diagnosed with heart failure with preserved ejection fraction using the 2016 European Society of Cardiology’s guideline on heart failure, including [27]:
  ○ Signs and symptoms of heart failure:
    ▪ Symptoms: dyspnea, orthopnea, paroxysmal nocturnal dyspnea, ankle swelling, reduced exercise capacity;
    ▪ Signs: elevated jugular venous pressure, hepatojugular reflux, third heart sound, and laterally displaced point of maximal impulse.
  ○ N-terminal pro-brain natriuretic peptide (NT-proBNP) ≥ 300 pg/mL in acute setting or ≥125 pg/mL in chronic setting;
  ○ Echocardiography with left ventricular ejection fraction (LVEF) ≥ 50% and at least one of these following criteria:
    ▪ Structural changes indicated by either left ventricle (LV) hypertrophy (any of the following: LV mass index ≥ 115 g/m^2^ in male or ≥95 g/m^2^ in female) or left atrium enlargement (LAE) (left atrial volume index ≥ 34 mL/m^2^);
    ▪ Diastolic dysfunction (E/e’ > 13 or e’ average < 9 cm/s).
• Agree to participate in the study and report outcomes for a 12-month period.

*Exclusion criteria:*

- Hospitalization due to cardiovascular disease in the preceding 30 days;
- Having a co-existing disease with a life expectancy < 1 year, as per the investigator’s judgement;
- Listed for a heart transplant;
- Primary stage D valvular heart disease requiring surgery/intervention or having a prosthetic/mechanical valve;
- Severe, unrepaired pericardiac disease;
- Complex, unrepaired congenital heart disease;
- Takotsubo disease, peripartum cardiomyopathy, chemotherapy-induced cardiomyopathy, cardiac sarcoidosis/amyloidosis;
- End-stage renal dysfunction, defined as persistent estimated glomerular filtration rate for at least 3 months (eGFR) < 15 mL/min (CKD-EPI Chronic Kidney Disease Epidemiology Collaboration Equation) or requiring renal replacement therapy;
- Child–Pugh–Turcotte C;
- Chronic obstructive pulmonary disease or any severe pulmonary disease requiring home oxygen;
- Pregnancy or lactation;
- Concurrent enrolment in another interventional device or drug trial;
- Refuse to participate in the study and report primary endpoints periodically for 12 months.

After agreeing to participate in the study, data on demographics, biomarkers, echocardiography, comorbidities, and medications were collected on the day of recruitment. Atrial fibrillation (AF) was confirmed with a 12-lead ECG. Peripheral artery disease (PAD) was diagnosed with the ankle brachia index, Doppler ultrasound, computerized tomography angiography, or angiogram. Coronary artery disease (CAD) was confirmed with the presence of a positive angiogram, non-invasive imaging, or stress test. Diagnosis of chronic kidney disease (CKD) adhered to the 2012 guideline of KDIGO (Kidney Disease: Improving Global Outcomes), which required a documented structural or functional abnormality persisting for at least 3 months. Chronic obstructive pulmonary disease (COPD) and asthma were diagnosed with a positive pulmonary function test, prior diagnosis, or current treatment.

Each echocardiography was performed, interpreted, and agreed upon by two qualified cardiologists at each recruitment site. The ejection fraction was calculated based on the biplane Simpson method. The left ventricular mass was calculated using the Devereux and Reichek cube formula and then indexed by body surface area to generate the left ventricular mass index (LVMI). The relative wall thickness (RWT) was calculated as twice the posterior wall thickness during diastole (PWTd) divided by the left ventricular internal diameter end diastole (LVIDD). The LV geometry was classified into 4 different categories depending on the presence of left ventricular hypertrophy (LVH) and whether RWT was greater than 0.42 (Supplementary Materials Table S1).

After the recruitment period, patients would then continue with the treatment algorithm in two teaching hospitals. These hospitals were tertiary, multispecialty medical facilities which had similar treatment protocols in alignment with Vietnam’s national guidelines. Patients were required to report outcomes for the upcoming 12 months. The primary outcome was time to the first event of all-cause mortality or hospitalization for heart failure. Any events reported would be adjudicated by the main investigator. Initially, follow-up was supposed to be performed every month for 12 months. However, due to prolonged lockdown, some patients were followed up every 3 months instead. Most follow-ups were performed through direct clinical consultation. For those who could not travel to the city center, the investigators contacted them by telephone to acquire information on symptoms, prescriptions, and outcomes.

**Figure 1:**
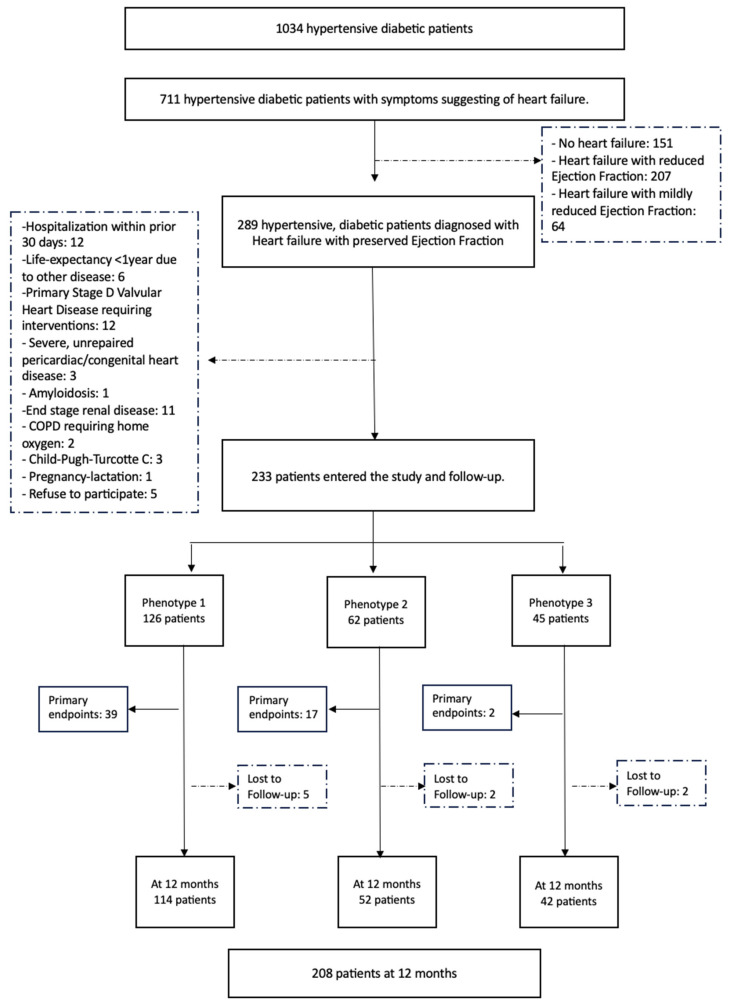
Flowchart of study recruitment and follow-up.

The study protocol is detailed in the flow chart below (Figure 1).

### 2.2. Statistical Analysis

We used Python (version 3.11) for data analysis and R for latent class analysis (LCA). LCA was performed to identify different phenotypes based on age ≥ 65, sex, comorbidities (obesity, AF, CAD, stroke/transient ischemic attack, PAD, dyslipidemia, CKD, COPD/asthma, anemia), and concentric hypertrophy (CH). These variables were clinically chosen after careful consideration of disease modeling in Vietnam as well as previous Asian studies phenotyping HFpEF patients. All variables were used as dichotomous input. When conducting LCA, there was a variety of classifications with different numbers of phenotypes. The optimal number was determined using Akaike’s Information Criterion (AIC) and Bayesian Information Criterion (BIC) values.

To provide information on internal robustness, we constructed a machine-learning model to classify patient phenotypes. Specifically, we used the DecisionTreeClassifier object in the scikit-learn package to create a machine-learning model where features were the ones used for LCA analysis, and labels were LCA-derived clustering information. We performed 10-fold cross-validation and reported the mean accuracy with standard deviation. The decision tree of the best fold was visualized using the plot_tree function.

Mean and median were used in the report of variables with normal and skewed distribution, respectively. Categorical variables were presented as percentages. We applied an ANOVA test and Mood’s median test for continuous variables, depending on the number of variables and pattern of distribution. The Chi-square test was used for categorical ones. For comparison of the primary outcome among phenotypes, Kaplan–Meier curves with log-rank testing were plotted to compare the event-free survival at any specific point of time during the 12-month follow-up.

For treatment response, because our study was observational, we employed the inverse probability of treatment weighting (IPTW) to statistically balance the confounders between patients with and without treatment. In IPTW, weights were given for everyone in the treated groups (propensity score) and untreated groups 1/(1-propensity score). By doing that, the treated individuals with a lower probability of treatment would have a larger weight and consequently balance their influence with those who had a higher probability of treatment (“standardization”). However, as we put a weight on each patient, an individual could be present more than once, thereby expanding the original population to form a new “pseudo-population”. In our study, each weight was carefully chosen based on the existing literature and clinical expertise. They included age, sex, obesity, NTproBNP level, AF, CKD, CAD, anemia, LAE, LVH, RAAS inhibitor, beta blocker, MRA, SGLT2 inhibitor, and furosemide. Weighted Kaplan–Meier curves for different event-free survival with specific treatments (RAAS inhibitor, beta blocker, MRA, and SGLT2 inhibitor) were presented separately for each phenotype. These four medications were chosen based on established evidence of reducing composite outcomes in HFpEF patients (EMPEROR-Preserved and DELIVER for SGLT2 inhibitor) or certain subgroups (CHARM-Preserved and PEP-CHF for RAAS inhibitor, TOPCAT for MRA). All tests were two-tailed and considered statistically significant when *p* < 0.05. To account for multiple testing, we employed the Benjamini–Hochberg procedure to adjust *p*-values.

## 3. Results

In total, 233 patients were included in the study. The mean age was 73. 67.8% of patients were female and 28.3% were obese. The two most common comorbidities were dyslipidemia (99.6%) and CAD (77.3%). The median EF was 61.0% and the median NTproBNP was 866 pg/mL. 66.5% of patients had LAE and 56.7% had LVH. The prescription rates of RAAS inhibitor, beta blocker, MRA, and SGLT2 inhibitor were 76.8%, 69.5%, 23.6%, and 18.5%, respectively.

Using latent class analysis, three metabolic phenotypes were identified. We selected n = 3 as the optimal number of phenotypes since the three-class model achieved the best and second-best result for AIC and BIC criteria, respectively. Supplementary Table S2 presented the fit statistics of each model. To facilitate the classification of patients into each phenotype, we provided our decision tree model with an accuracy of 0.93 (0.05) in Supplementary Figure S2.

Baseline patient characteristics, comorbidities, and investigations of each phenotype were detailed in Table 1. Phenotype 1 (n = 126) included lean, elderly females with the highest comorbidity burden, especially CKD, anemia, CAD, and CH. Phenotype 2 (n = 62) consisted of younger males with CAD. They were most likely to have a history of myocardial infarction but least likely to have AF. Phenotype 3 (n = 45) depicted obese, elderly patients with the highest rate of AF and stroke, the largest left atrial volume, and the lowest rate of CKD.

After 12 months, the composite endpoint of all-cause mortality and heart failure hospitalization was observed in 24.9% of patients. A total of 3.9% were lost to follow-up.

**Figure 2:**
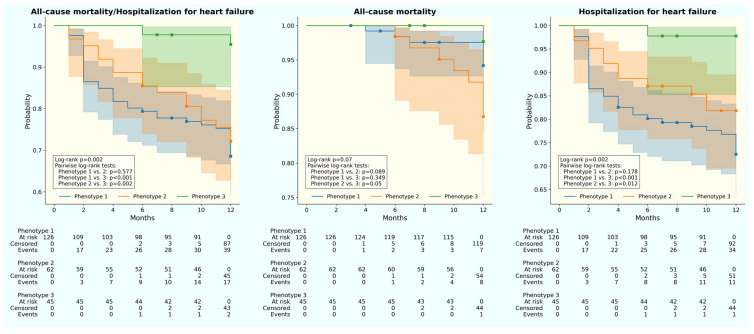
Kaplan–Meier curve for 12-month outcomes in three hypertensive-diabetic HFpEF phenotypes. (**Left**) All-cause mortality and hospitalization for hear failure. (**Middle**) All-cause mortality. (**Right**) Hospitalization for heart failure. Blue: phenotype 1; orange: phenotype 2; green: phenotype 3.

Among the three phenotypes, phenotype 1 and 2 were associated with higher all-cause mortality and HHF (*p* = 0.002) (Figure 2 (Left)). The difference was mostly observed in HHF (*p* = 0.002) (Figure 2 (Right)) rather than all-cause mortality (*p* = 0.070) ((Figure 2 (Middle)). Specifically, phenotype 1 experienced more HHF (27.2% vs. 17.7%), while phenotype 2 had more all-cause mortality (12.9% vs. 5.6%).

**Figure 3:**
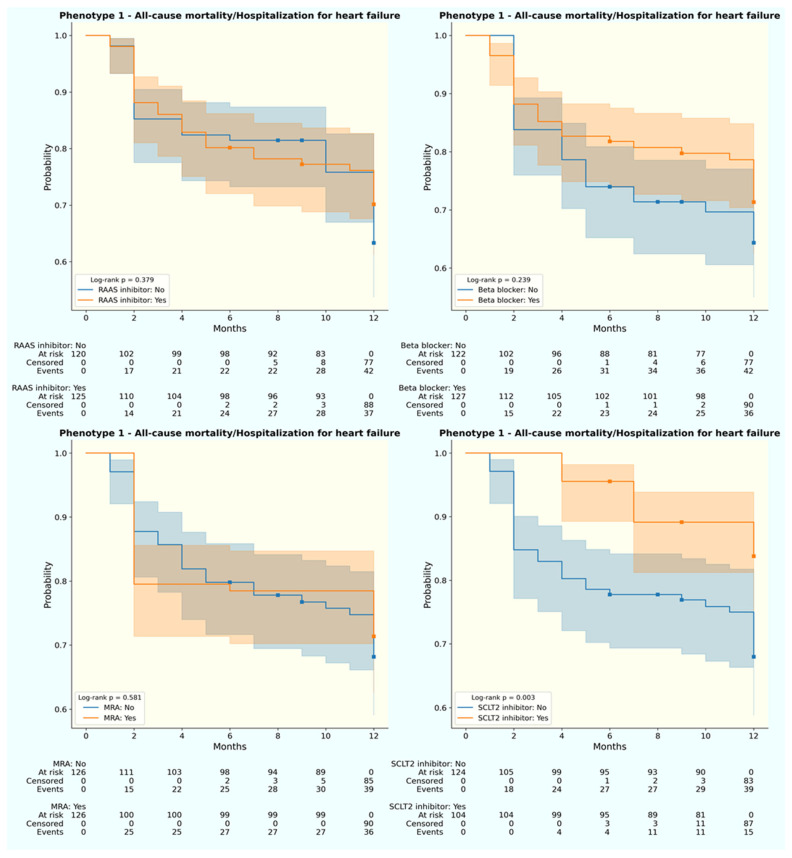
Weighted Kaplan–Meier curve for 12-month primary outcome by treatment status of phenotype 1. (**Upper left**) RAAS inhibitor; (**upper right**) beta blocker; (**lower left**) MRA; (**lower right**) SGLT2 inhibitor. Blue: without medication; orange: with medication.

**Figure 4:**
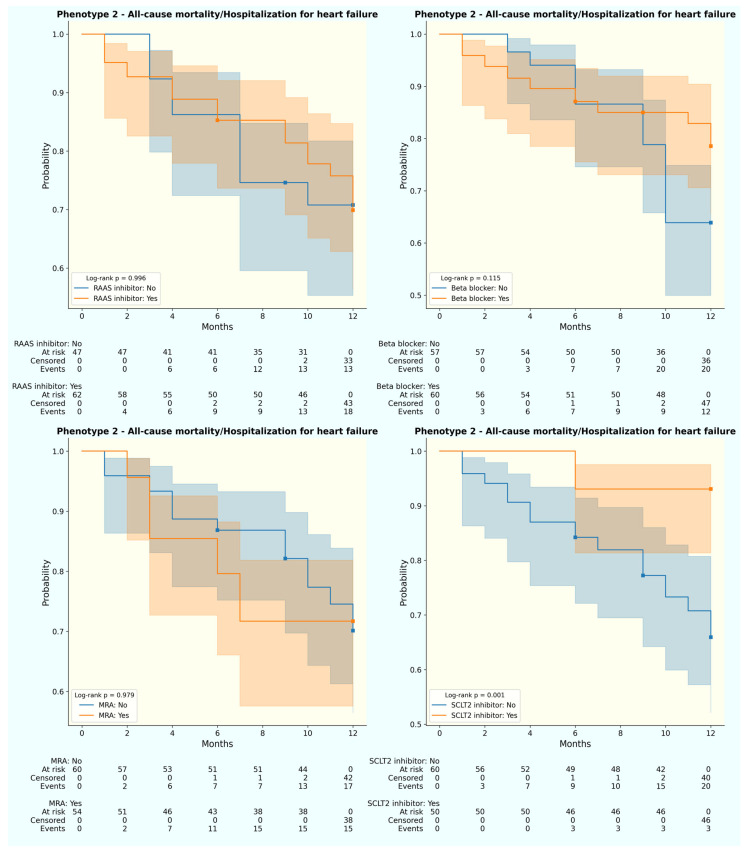
Weighted Kaplan–Meier curve for 12-month primary outcome by treatment status of phenotype 2. (**Upper left**) RAAS inhibitor; (**upper right**) beta blocker; (**lower left**) MRA; (**lower right**) SGLT2 inhibitor. Blue: without medication; orange: with medication.

Not just the 12-month primary outcome, disparities were also observed in treatment responses across phenotypes, despite statistically similar prescription rates (Table 1). For the primary endpoint, SGLT2 inhibitor was associated with better all-cause mortality and HHF for phenotype 1 (log-rank *p* = 0.003) and 2 (log-rank *p* = 0.001) (Figure 3 and Figure 4). In terms of each separate endpoint, individuals from phenotype 2 underwent fewer HHF with beta-blocker use (log-rank *p* = 0.011). Meanwhile, patients from phenotype 1 experienced fewer all-cause mortality with RAS inhibitor uptake (log-rank *p* = 0.003) or beta blocker prescription (log-rank *p* = 0.024). No therapy was associated with better outcomes in phenotype 3 (Supplementary Figure S1).

## 4. Discussion

Our study added three important findings to the growing body of HFpEF literature. First, among hypertensive-diabetic HFpEF, there existed three distinct phenotypes, which could be easily identified with simple clinical parameters, leading to easy application in practice. Second, these phenotypes were associated with different all-cause mortality and hospitalization for heart failure. The “AF-predominant” group (phenotype 3) experienced the least events as opposed to the other two despite old age and a high number of comorbidities. Third, each phenotype responded differently to a specific HFpEF therapy, including RAAS inhibitor, beta blocker, and SGLT2 inhibitor.

Our study was conducted in two tertiary, multispecialty hospitals with large patient volumes, which enabled us to include a broad range of HFpEF patients for phenotyping. Applying common clinical parameters (age, sex, comorbidities, and CH), we identified three different HFpEF phenotypes in the hypertensive-diabetic umbrella. On the one hand, these phenotypes mirrored the typical ones described in the general HFpEF population. The four most common phenotypes in HFpEF trials were “garden-variety”, “CAD-associated”, “AF-predominant”, and “right heart failure-predominant” [15,16,19,28]. Phenotype 1, with the highest rates of comorbidities, was similar to the “garden-variety” cluster. Phenotype 2, with the highest rate of ischemia and previous myocardial infarction, was identical to the “CAD-associated” group. Phenotype 3, with the highest rate of AF, was suggestive of the “AF-predominant” bracket. On the other hand, these three phenotypes reflected the distinct pathophysiology of hypertensive-diabetic cardiomyopathy. One hypothesis for diabetes-induced HFpEF was the expansion and inflammation of epicardial tissue, leading to microvascular dysfunction of the coronary vascular bed, proinflammatory states, and cardiac fibrosis [29]. These phenomena could also be observed in the atrial myopathy mechanism and the hemodynamic pathway of hypertension-induced cardiomyopathy [30,31,32]. Therefore, the co-prevalence of hypertension, diabetes, and AF further increases the risk of incident HFpEF, as seen in phenotype 3. Similarly, because hypertension, diabetes, and CAD share the same mechanism of inflammation, oxidative stress, neurohormonal activation, microvascular and vascular dysfunction, they subsequently stiffen cardiomyocytes, leading to the development of HFpEF [33,34,35]. Hence, CAD, one common comorbidity of HFpEF, can be observed more frequently in a subgroup of hypertensive-diabetic HFpEF, as seen with phenotype 2 and 1. In phenotype 1, however, a higher comorbidity burden was also noted, especially CKD and CH. Hallmarks of CKD-associated cardiomyopathy included LVH and profound cardiac fibrosis on histology [36]. As hypertension and diabetes played key roles in the development of both HFpEF and CKD, their co-existence further increases the prevalence of CKD in HFpEF, which consequently worsens prognosis, as seen in phenotype 1 [36,37,38].

The most striking finding of our work was the phenotype-specific outcomes. Lower 12-month all-cause mortality and heart failure hospitalization were noted in phenotype 3 as opposed to other groups. AF was increasingly prevalent in the elderly population and was associated with worse outcomes in HFpEF, including stroke [39]. However, a recent study on the Asian population revealed that the “rhythm trouble” phenotype had the most favorable prognosis among HFpEF phenotypes [13]. In contrast, phenotype 1 and 2 experienced higher rates of mortality and hospitalization within the first 12 months. The majority of these patients had CAD and a history of myocardial infarction. The rising prevalence of CAD, as well as its association with higher cardiovascular mortality, has been demonstrated in HFpEF trials [40,41,42]. In our study with hypertensive-diabetic patients, CAD was the predominant comorbidity in all phenotypes. Yet, prior myocardial infarction mainly occurred in phenotype 1 and 2, which can partially explain their higher composite outcome. In phenotype 1, apart from CAD, the co-prevalent CKD (47.6%), anemia (87.3%), LVH (58.7%), and concentric hypertrophy (50.0%) further heightened the risk of cardiovascular events. The combination of CKD, LVH, DM, and HTN was usually observed in the phenotype with the worst outcome as in TOPCAT trial (phenogroup 3) or ASIAN-HF registry (Lean Diabetes) [11,25]. Phenotype 1 also had the highest rate of anemia, which had been suggested to hold prognostic value according to emerging evidence from contemporary HFpEF trials [43]. The rate of LVH and CH were higher in phenotype 1 (56.7% and 40.3%) in contrast to diabetic HFpEF patients in the ASIAN-HF (46.4% and 31.0%) [44]. Therefore, phenotype 1, typified by elderly patients with high comorbidity burden, especially LVH and CH, was associated with the worst outcome among the three groups. In fact, the rate of the composite event of our study was generally higher than that of ASIAN-HF (24.9% vs. 14.8%) [45]. The difference was more pronounced in heart failure hospitalization (19.7% vs. 10.6%) as opposed to all-cause mortality (6.9% vs. 5.7%) [45]. Compared to the ASIAN-HF population, our patients were older, leaner, and more often female, with a higher burden of comorbidity. Comorbidities are common drivers for heart failure, either by direct cardiac insults in HFrEF or chronic inflammation in HFpEF [46]. In an era of an aging HFpEF population, increasing comorbidity burden was associated with worsened prognosis, which could be related to polypharmacy, drug interaction, and hindrance in initiating and optimizing heart failure therapy [47,48,49].

While SGLT2 inhibitor is the cornerstone of treatment for HFpEF patients, the high burden of comorbidity often requires additional therapy [18,50]. Our study suggested the importance of the phenotypic approach in further improving cardiovascular outcomes in hypertensive-diabetic HFpEF patients. While phenotype 1 and 2 reported the highest composite outcomes, they were more likely to respond to SGLT2 inhibitor. This medication was associated with fewer composite outcomes for both phenotype 1 (*p* = 0.002) and phenotype 2 (*p* = 0.001). When we took into consideration all-cause mortality and HHF separately, phenotype 1 and 2 demonstrated different treatment responses with RAAS inhibitor and beta blocker. For phenotype 1 with high burden of CKD and LVH, RAS inhibitor was associated with fewer mortality. The presence of CKD or LVH increases adverse cardiovascular events in diabetic individuals, as well as in HFpEF patients [51,52,53,54]. These two conditions, CKD and LVH, are often co-existing in CKD-associated cardiomyopathy [36,51]. RAAS inhibitor is the treatment of choice both to reverse LVH and to delay the progression of CKD [5,50]. This was in keeping with our findings that RAAS inhibitors was associated with fewer mortality in phenotype 1. Similarly, in phenotype 1 and 2 with high rates of previous myocardial infarction, fewer events were observed in patients taking beta blocker. Even though beta blocker did not have an indication for HFpEF in general, the rate of beta blocker intake was high in most trials (86% in EMPEROR-Preserved, 76% in DELIVER), reflecting the comorbidity-driven indication, especially CAD and tachycardia [6,7,55]. While long-term use of beta blocker did not provide additional protection after 3 years post-myocardial infarction in patients with “normal” ejection fraction, its benefit on patients with HFpEF was controversial [55,56,57]. A recent meta-analysis from randomized controlled trials demonstrated that in HFpEF patients with comorbid CAD, the use of beta blocker led to improvement in exercise capacity [58]. Beta blockers enhanced myocardial perfusion in ischemic patients by reducing myocardial ischemia and prolonging diastolic filling time. In our study, CAD was present in 77.3% of patients, with the highest history of myocardial infarction in phenotype 2 and 1. The use of beta blocker in these two phenotypes, therefore, was associated with better cardiovascular prognoses. In our study, the SGLT2 inhibitor did not display protection against combined endpoints in phenotype 3, which could be related to the small population (n = 45) and low prescription rate of SGLT2 inhibitor (17.8%).

All in all, our study implied an important message in clinical practice. Hypertensive-diabetic patients accounted for a large proportion of HFpEF and were notoriously associated with worse prognoses. Yet, even though previous studies had reported conflicting findings in their presentations and outcomes, hypertensive-diabetic patients were altogether pigeonholed as “metabolic” phenotype. We demonstrated for the first time that among this seemingly homogeneous population existed heterogenous phenotypes with different clinical features, 12-month outcomes, and treatment responses. This finding suggested hypertensive-diabetic patients would benefit from phenotype-based risk stratification and personalized management.

Our study had three main limitations. First, the prescription rate of SGLT2 inhibitor was low because the indication of this medication for HFpEF was only approved by the Ministry of Health in Vietnam from July 2022. During the recruitment period from January 2021 to April 2022, the use of SGLT2 inhibitor was subjective to treating physicians. We reported a similar rate of SGLT2 inhibitor uptake (18.5%) with the Swedish Heart Failure registry (12.5%) [59]. Another limitation of our study was related to the prolonged COVID-19 lockdown in 2021. During this period, we could not perform monthly follow-up on every patient. Instead, some of our patients returned to the clinics every 3 months. Two patients from phenotype 1 did report hospitalizations during this 3-month interval. However, without sufficient evidence (i.e., discharge paper, discharge prescription), these patients’ self-report events could not be adjudicated; thus, they were not counted. Finally, our study was initiated before the success of EMPEROR-Preserved and DELIVER, and no life-saving therapy for HFpEF was proven during most of our recruitment period. As such, our study was designed and carried out as an observational one. When assessing treatment responses, we employed the IPTW and provided weights to potential confounders so as to improve the non-randomized nature of the observational cohort. Yet more large-scale, multicentered, randomized controlled studies with prolonged follow-up should be carried out for better evaluation of phenotype profiling, long-term outcome, and treatment response.

## 5. Conclusions

Our novel study described the modern-day population of hypertensive-diabetic HFpEF. They were lean, elderly females with high comorbidity burden who experienced a high incidence of all-cause mortality and hospitalization for heart failure. While hypertension and diabetes are common comorbidities with heightened cardiovascular risk in HFpEF, not all hypertensive-diabetic patients carry the same prognosis. We determined for the first time three distinct hypertensive-diabetic HFpEF phenotypes with different 12-month composite outcomes and treatment responses, necessitating the need for a personalized approach to optimize patient care.

## Figures and Tables

**Table 1:** Baseline patient characteristics on demographics, comorbidities, and investigations in three hypertensive-diabetic phenotypes.

		All Patients			
	Total (n = 233)	Phenotype 1 (n = 126)	Phenotype 2 (n = 62)	Phenotype 3 (n = 45)	*p* *
Demographics					
Age year (SD)	75 (10)	75 (10)	68 (10)	74 (10)	**<0.001**
Female n (%)	158 (67.8)	126 (100)	10 (16.1)	22 (48.9)	**<0.001**
BMI kg/m^2^ (IQR)	23.3 (21.3; 25.8)	23.0 (21.1; 25.0)	23.4 (21.5; 25.8)	24.0 (21.6; 26.7)	0.544
Obesity n (%)	66 (28.3)	31 (24.6)	17 (27.4)	18 (40)	0.312
SBP mmHg (IQR)	130.0 (120.0; 140.0)	130.0 (115.0; 140.0)	130.0 (120.0; 145.0)	130.0 (125.0; 140.0)	0.855
DBP mmHg (IQR)	75.0 (70.0; 80.0)	70.0 (70.0; 80.0)	70.0 (65.0; 80.0)	80.0 (70.0; 80.0)	0.152
Heart rate bpm (IQR)	78.0 (70; 85.0)	78.0 (72.0; 85.0)	79.0 (70.0; 84.0)	75.0 (70.0; 85.0)	0.855
**Comorbidities**					
CAD n (%)	180 (77.3)	93 (73.8)	54 (87.1)	33 (73.3)	0.229
Prior MI n (%)	89 (32.8)	37 (29.4)	45 (72.6)	7 (15.6)	**<0.001**
AF n (%)	68 (29.2)	24 (19.1)	1 (1.6)	43 (95.6)	**<0.001**
Stroke/TIA n (%)	25 (10.7)	10 (7.9)	7 (11.3)	8 (17.8)	0.357
PAD n (%)	20 (8.6)	12 (9.5)	5 (8.1)	3 (6.7)	0.855
COPD/asthma n (%)	21 (9.0)	6 (4.8)	9 (14.5)	6 (13.3)	0.144
CKD n (%)	89 (38.2)	60 (47.6)	19 (30.7)	10 (22.2)	**0.0** **17**
Anemia n (%)	149 (64.0)	110 (87.3)	24 (38.7)	15 (33.3)	**<0.001**
Dyslipidemia n (%)	232 (99.6)	125 (99.2)	62 (100)	45 (100)	0.798
≥4 comorbidities (including hypertension, diabetes) n (%)	191 (82.0)	115 (91.3)	37 (59.7)	39 (86.7)	**<0.001**
**Investigations**					
LDL-c mmol/L (IQR)	2.3 (1.8; 3.2)	2.3 (1.8; 3.2)	2.5 (1.9; 3.2)	1.9 (1.6; 2.8)	0.480
Triglyceride mmol/L (IQR)	1.7 (1.2; 2.4)	1.8 (1.2; 2.5)	1.7 (1.2; 2.5)	1.4 (1.2; 2.1)	0.129
HDL-c (IQR)	1.1 (0.9; 1.2)	1.1 (0.9; 1.2)	1.1 (0.9; 1.1)	1.0 (0.8; 1.2)	0.968
Total cholesterol (IQR)	3.9 (3.3; 4.9)	3.8 (3.3; 4.9)	4.1 (3.4; 4.9)	3.7 (2.7; 4.4)	0.357
HbA1c % (IQR)	7.5 (6.6; 8.8)	7.5 (6.7; 8.8)	7.4 (6.5; 9.0)	7.3 (6.6; 8.6)	0.581
NTproBNP pg/mL (IQR)	866.0 (313.0; 3000.0)	763.5 (282.0; 3634.0)	821.0 (342.3; 2466.5)	1418.0 (643.0; 2208)	0.480
EF % (IQR)	61.0 (56.0; 66.0)	61.5 (57.0,64.0)	60 (55.0; 64.0)	59.0 (56.0; 63.0)	0.855
LVH n (%)	132 (56.7)	74 (58.7)	37 (59.7)	21 (46.7)	0.480
CH n (%)	94 (40.3)	63 (50.0)	20 (32.3)	11 (24.4)	**0.0** **17**
LAE n (%)	155 (66.5)	74 (58.7)	43 (69.4)	38 (84.4)	**0.0** **22**
E/e’ > 9 n (%)	220 (94.4)	117 (92.9)	53 (85.5)	44 (97.8)	0.152
**Treatments**					
SGLT2 inhibitor n (%)	43 (18.5)	19 (15.1)	16 (25.8)	8 (17.8)	0.370
RAAS inhibitor n (%)	179 (76.8)	95 (75.4)	46 (74.2)	38 (84.4)	0.545
Beta blocker n (%)	162 (69.5)	82 (65.1)	46 (74.2)	34 (75.6)	0.476
MRA n (%)	55 (23.6)	26 (20.6)	18 (29.0)	11 (24.4)	0.579
Furosemide n (%)	64 (27.5)	37 (29.4)	15 (24.2)	12 (26.7)	0.855

* Corrected with Benjamini–Hochberg procedure (false discovery rate 0.05); SD: standard deviation, IQR: interquartile range, BMI: body mass index, SBP: systolic blood pressure, DBP: diastolic blood pressure, bmp: beats per minute, CAD: coronary artery disease, MI: myocardial infarction, AF: atrial fibrillation, TIA: transient ischemic attack, PAD: peripheral artery disease, COPD: chronic obstructive pulmonary disease, CKD: chronic kidney disease, EF: ejection fraction, LVH: left ventricular hypertrophy, CH: concentric hypertrophy; LAE: left atrial enlargement, SGLT2 inhibitor: sodium–glucose cotransporter-2 inhibitor, RAAS inhibitor: renin–angiotensin–aldosterone inhibitor, MRA: mineralocorticoid receptor antagonist.

## Data Availability

The data presented in this study are not publicly available because they are one part of an ongoing Ph.D. project. Some data could be available upon reasonable written request to the corresponding author, H.N.C.

## Supplementary Materials

The following supporting information can be downloaded at: https://www.mdpi.com/article/10.3390/jpm13081218/s1, Table S1: Geometry classification of left ventricle, Table S2: Fit Statistic Information on LCA analysis, Figure S1: Weighted Kaplan–Meier curve for 12-month primary outcome by treatment status of phenotype 3, Figure S2: Decision tree for phenotype assignment.
