# Supplementary material for: Phenotype-Specific Outcome and Treatment Response in Heart Failure with Preserved Ejection Fraction with Comorbid Hypertension and Diabetes: A 12-Month Multicentered Prospective Cohort Study
Source: J Pers Med. 2023 Jul 31;13(8):1218. doi: 10.3390/jpm13081218 (PMC10455077; doi:10.3390/jpm13081218)
Supplement: Supplementary file 1 [file jpm-13-01218-s001.zip › Figure S1 - Supplementary Materials AProf Hoa Chau JPM 7.2023 .pdf]

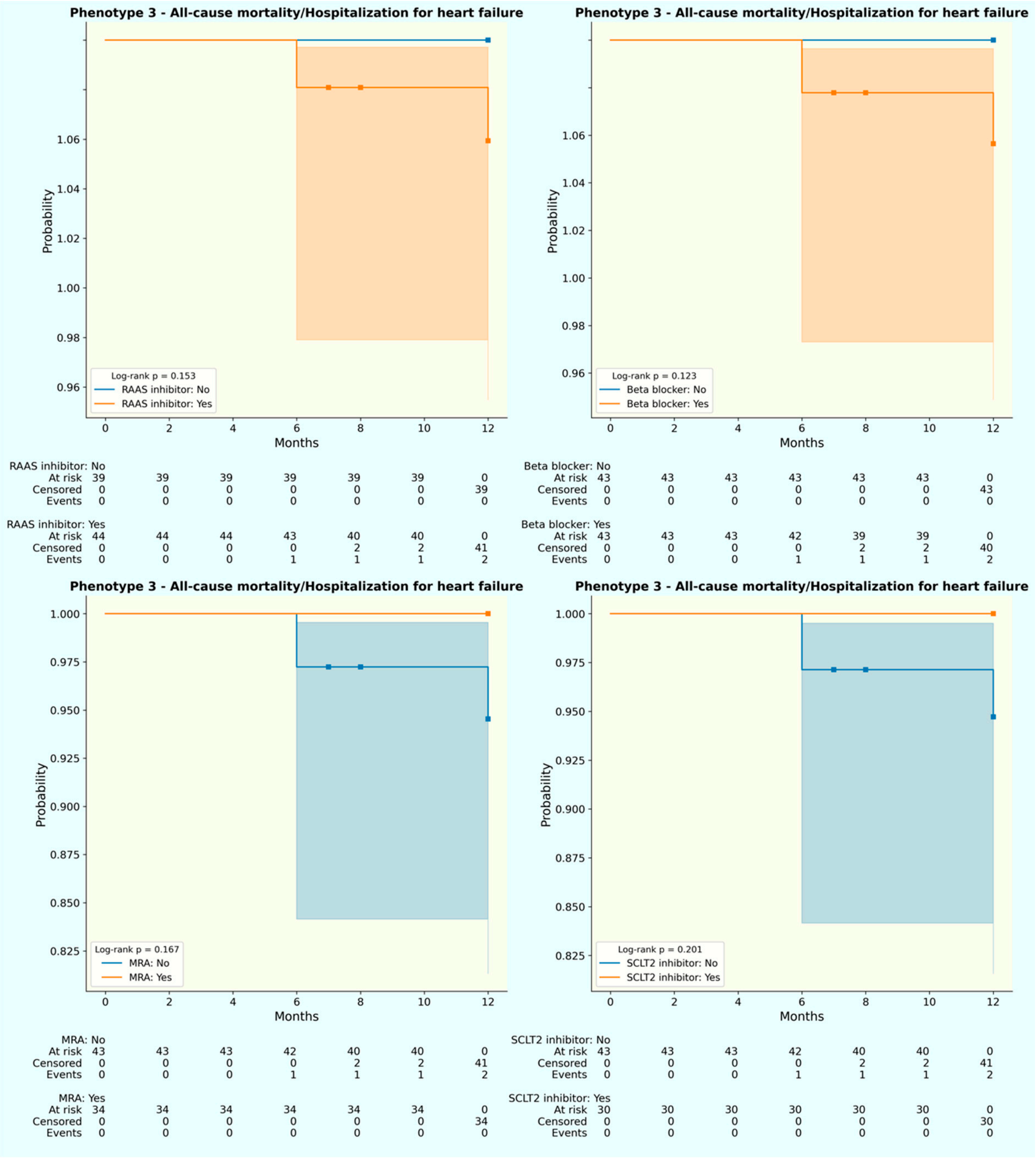

Figure S1. Weighted Kaplan-Meier curve for 12-month primary outcome by treatment status of phenotype 3. (**Upper Left**) RAAS inhibitor; (**Upper Right**) Beta blocker; (**Lower Left**) MRA; (**Lower Right**) SGLT2 inhibitor.

Blue: without medication; Orange: with medication
