# Supplementary material for: Phenotype-Specific Outcome and Treatment Response in Heart Failure with Preserved Ejection Fraction with Comorbid Hypertension and Diabetes: A 12-Month Multicentered Prospective Cohort Study
Source: J Pers Med. 2023 Jul 31;13(8):1218. doi: 10.3390/jpm13081218 (PMC10455077; doi:10.3390/jpm13081218)
Supplement: Supplementary file 1 [file jpm-13-01218-s001.zip › Figure S2 - Supplementary Materials AProf Hoa Chau JPM 7.2023 .pdf]

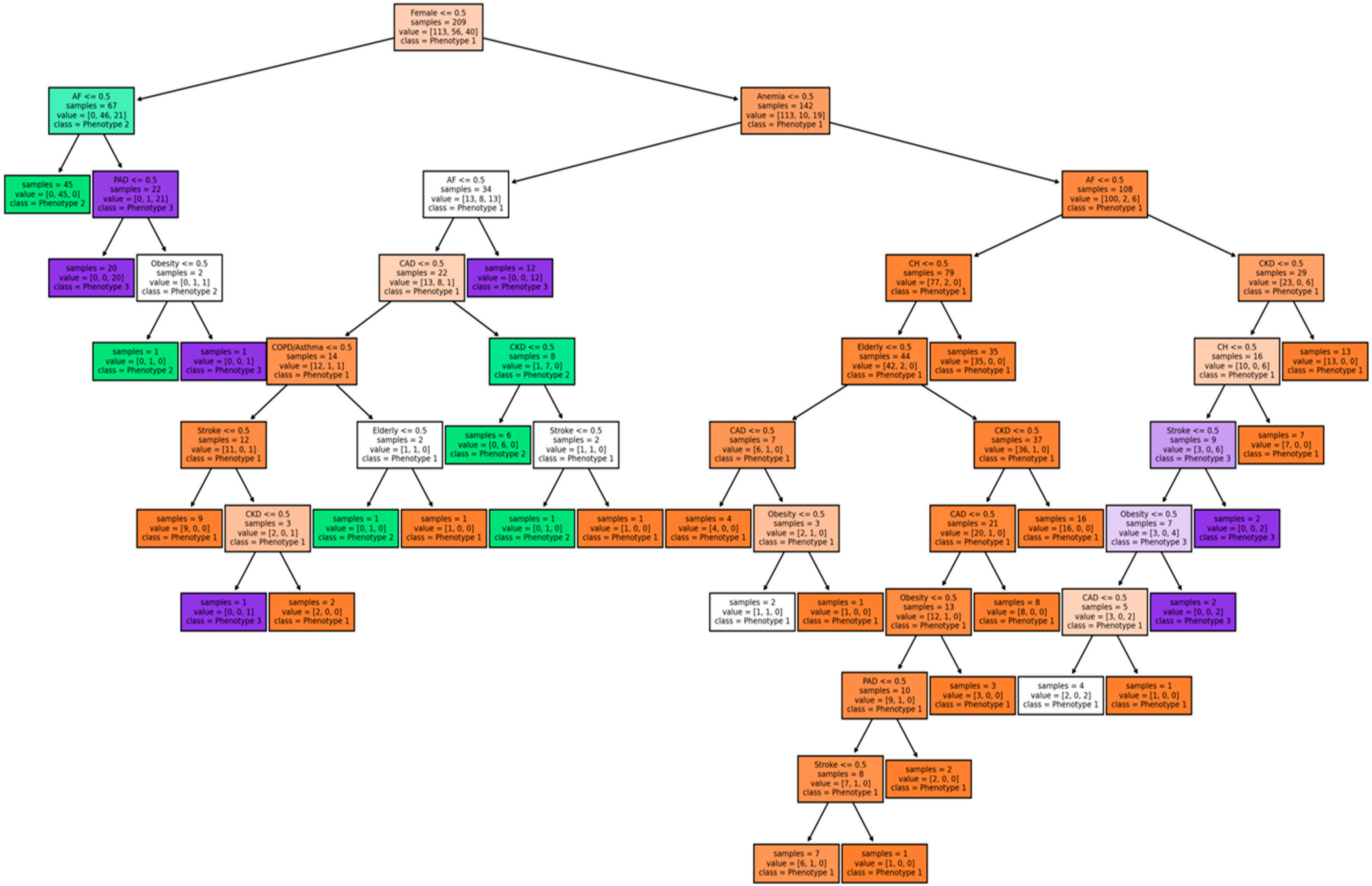
Female <= 0.5 samples = 209 value = [113, 56, 40] class = Phenotype 1
AF <= 0.5 samples = 67 value = [0, 46, 21] class = Phenotype 2
Anemia <= 0.5 samples = 142 value = [113, 10, 19] class = Phenotype 1
samples = 45 value = [0, 45, 0] class = Phenotype 2
PAD <= 0.5 samples = 22 value = [0, 1, 21] class = Phenotype 3
AF <= 0.5 samples = 34 value = [13, 8, 13] class = Phenotype 1
AF <= 0.5 samples = 108 value = [100, 2, 6] class = Phenotype 1
samples = 20 value = [0, 0, 20] class = Phenotype 3
Obesity <= 0.5 samples = 2 value = [0, 1, 1] class = Phenotype 2
CAD <= 0.5 samples = 22 value = [13, 8, 1] class = Phenotype 1
samples = 12 value = [0, 0, 12] class = Phenotype 3
CH <= 0.5 samples = 79 value = [77, 2, 0] class = Phenotype 1
CKD <= 0.5 samples = 29 value = [23, 0, 6] class = Phenotype 1
samples = 1 value = [0, 1, 0] class = Phenotype 2
samples = 1 value = [0, 0, 1] class = Phenotype 3
COPD/Asthma <= 0.5 samples = 14 value = [12, 1, 1] class = Phenotype 1
CKD <= 0.5 samples = 8 value = [1, 7, 0] class = Phenotype 2
Elderly <= 0.5 samples = 44 value = [42, 2, 0] class = Phenotype 1
samples = 35 value = [35, 0, 0] class = Phenotype 1
CH <= 0.5 samples = 16 value = [10, 0, 6] class = Phenotype 1
samples = 13 value = [13, 0, 0] class = Phenotype 1
Stroke <= 0.5 samples = 12 value = [11, 0, 1] class = Phenotype 1
Elderly <= 0.5 samples = 2 value = [1, 1, 0] class = Phenotype 1
samples = 6 value = [0, 6, 0] class = Phenotype 2
Stroke <= 0.5 samples = 2 value = [1, 1, 0] class = Phenotype 1
CAD <= 0.5 samples = 7 value = [6, 1, 0] class = Phenotype 1
CKD <= 0.5 samples = 37 value = [36, 1, 0] class = Phenotype 1
Stroke <= 0.5 samples = 9 value = [3, 0, 6] class = Phenotype 3
samples = 7 value = [7, 0, 0] class = Phenotype 1
samples = 9 value = [9, 0, 0] class = Phenotype 1
CKD <= 0.5 samples = 3 value = [2, 0, 1] class = Phenotype 1
samples = 1 value = [0, 1, 0] class = Phenotype 2
samples = 1 value = [1, 0, 0] class = Phenotype 1
samples = 1 value = [0, 1, 0] class = Phenotype 2
samples = 1 value = [1, 0, 0] class = Phenotype 1
samples = 4 value = [4, 0, 0] class = Phenotype 1
Obesity <= 0.5 samples = 3 value = [2, 1, 0] class = Phenotype 1
CAD <= 0.5 samples = 21 value = [20, 1, 0] class = Phenotype 1
samples = 16 value = [16, 0, 0] class = Phenotype 1
Obesity <= 0.5 samples = 7 value = [3, 0, 4] class = Phenotype 3
samples = 2 value = [0, 0, 2] class = Phenotype 3
samples = 1 value = [0, 0, 1] class = Phenotype 3
samples = 2 value = [2, 0, 0] class = Phenotype 1
samples = 2 value = [1, 1, 0] class = Phenotype 1
samples = 1 value = [1, 0, 0] class = Phenotype 1
Obesity <= 0.5 samples = 13 value = [12, 1, 0] class = Phenotype 1
samples = 8 value = [8, 0, 0] class = Phenotype 1
CAD <= 0.5 samples = 5 value = [3, 0, 2] class = Phenotype 1
samples = 2 value = [0, 0, 2] class = Phenotype 3
PAD <= 0.5 samples = 10 value = [9, 1, 0] class = Phenotype 1
samples = 3 value = [3, 0, 0] class = Phenotype 1
samples = 4 value = [2, 0, 2] class = Phenotype 1
samples = 1 value = [1, 0, 0] class = Phenotype 1
Stroke <= 0.5 samples = 8 value = [7, 1, 0] class = Phenotype 1
samples = 2 value = [2, 0, 0] class = Phenotype 1
samples = 7 value = [6, 1, 0] class = Phenotype 1
samples = 1 value = [1, 0, 0] class = Phenotype 1

Figure S2. Decision tree for phenotype assignment.

Samples and value indicate the number of data points and phenotypes at each node. Since all features are categorical data, “<= 0.5” indicates the absence of features.

Abbreviation: AF: Atrial Fibrillation, CAD: Coronary artery disease, CH: Concentric hypertrophy, CKD: Chronic kidney disease, COPD: Chronic obstructive pulmonary disease, PAD: Peripheral artery disease
