# Supplementary material for: Phenotype-Specific Outcome and Treatment Response in Heart Failure with Preserved Ejection Fraction with Comorbid Hypertension and Diabetes: A 12-Month Multicentered Prospective Cohort Study
Source: J Pers Med. 2023 Jul 31;13(8):1218. doi: 10.3390/jpm13081218 (PMC10455077; doi:10.3390/jpm13081218)
Supplement: Supplementary file 1 [file jpm-13-01218-s001.zip › Table S1- Supplementary Materials- AProf Hoa Chau JPM 7.2023.pdf]

Table S1. Geometry classification of Left Ventricle on Echocardiogram

Left Ventricular Mass Index	Relative Wall Thickness ≤0.42	Relative Wall Thickness >0.42
< 115g/m^2 (male) or < 95 g/m^2 (female)	Normal	Concentric Remodelling
≥ 115g/m^2 (male) or ≥ 95 g/m^2 (female)	Eccentric Hypertrophy	Concentric Hypertrophy
