# Supplementary material for: Phenotype-Specific Outcome and Treatment Response in Heart Failure with Preserved Ejection Fraction with Comorbid Hypertension and Diabetes: A 12-Month Multicentered Prospective Cohort Study
Source: J Pers Med. 2023 Jul 31;13(8):1218. doi: 10.3390/jpm13081218 (PMC10455077; doi:10.3390/jpm13081218)
Supplement: Supplementary file 1 [file jpm-13-01218-s001.zip › Table S2- Supplementary Materials- AProf Hoa Chau JPM 7.2023.pdf]

Table S2. Fit Statistic Information on LCA analysis

Model	BIC	AIC	Maximum log-likelihood
2-class	2423.71	2358.14	-1160.07
3-class	2446.46	2346.38	-1144.19
4-class	2485.23	2350.64	-1136.32
5-class	2525.30	2356.20	-1129.01
6-class	2550.57	2346.96	-1114.48
